# The Basophil IL-18 Receptor Precisely Regulates the Host Immune Response and Malaria-Induced Intestinal Permeability and Alters Parasite Transmission to Mosquitoes without Effect on Gametocytemia

**DOI:** 10.4049/immunohorizons.2200057

**Published:** 2022-08-19

**Authors:** Erinn L. Donnelly, Nora Céspedes, Gretchen Hansten, Delaney Wagers, Anna M. Briggs, Casey Lowder, Joseph Schauer, Lori Haapanen, Judy Van de Water, Shirley Luckhart

**Affiliations:** * Department of Biological Sciences, University of Idaho, Moscow, ID; † Department of Entomology, Plant Pathology and Nematology, University of Idaho, Moscow, ID; ‡ Division of Rheumatology, Allergy and Clinical Immunology, University of California, Davis, CA

## Abstract

We have recently demonstrated that basophils are protective against intestinal permeability during malaria and contribute to reduced parasite transmission to mosquitoes. Given that IL-18 is an early cytokine/alarmin in malaria and has been shown to activate basophils, we sought to determine the role of the basophil IL-18R in this protective phenotype. To address this, we infected control [*IL18r*^flox/flox^ or basoIL-18R (+)] mice and mice with basophils lacking the IL-18R [*IL18r*^flox/flox^ × Basoph8 or basoIL-18R (−)] with *Plasmodium yoelii yoelii* 17XNL, a nonlethal strain of mouse malaria. Postinfection (PI), intestinal permeability, ileal mastocytosis, bacteremia, and levels of ileal and plasma cytokines and chemokines were measured through 10 d PI. BasoIL-18R (−) mice exhibited greater intestinal permeability relative to basoIL-18R (+) mice, along with increased plasma levels of proinflammatory cytokines at a single time point PI, day 4 PI, a pattern not observed in basoIL-18R (+) mice. Surprisingly, mosquitoes fed on basoIL-18R (−) mice became infected less frequently than mosquitoes fed on basoIL-18R (+) mice, with no difference in gametocytemia, a pattern that was distinct from that observed previously with basophil-depleted mice. These findings suggest that early basophil-dependent protection of the intestinal barrier in malaria is mediated by IL-18, and that basophil IL-18R–dependent signaling differentially regulates the inflammatory response to infection and parasite transmission.

## INTRODUCTION

Malaria results from infection with parasites in the genus *Plasmodium* transmitted by *Anopheles* mosquitoes. Despite global malaria eradication efforts, the World Health Organization reported 627,000 deaths and 241 million cases in 2020 (1). Concomitant bacteremia is a complication that contributes to morbidity and mortality associated with malaria across age groups and clinical disease severities (2-5). Our previous studies with mouse models demonstrated that malaria-induced bacteremia arises from early intestinal mast cell (MC) influx or mastocytosis (6-8). The appearance of malaria-induced intestinal permeability to enteric bacteria, a phenomenon referred to as “leaky gut,” is preceded by a type 2–biased host immune response, specifically by the appearance of IL-4, IL-10, MC protease 1 (Mcpt1), and Mcpt4, as well as increased circulating basophils and eosinophils (8).

We previously showed that basophil-depleted mice exhibited significantly increased intestinal permeability at days 4, 6, and 8 postinfection (PI) with *Plasmodium yoelii yoelii* 17XNL compared with nondepleted mice (9). Further, basophil-depleted mice exhibited an increase in ileal MC numbers at 8 d PI, suggesting that in the context of malaria, basophils blunt increases in intestinal permeability and mastocytosis. Despite these differences, basophil-depleted and nondepleted mice had similar patterns of bacterial 16S DNA in blood that tracked with rising parasitemia. Network analyses of cytokines and chemokines revealed differences in the immune response to bacteria between genotypes that suggested that basophil-depleted mice were better able to control bacterial translocation at the level of the intestine, while the systemic response was more effective than the intestinal response in controlling bacterial translocation in nondepleted mice (9). Basophil depletion was also associated with increased gametocytemia and increased infection success in the mosquito host *Anopheles stephensi*, suggesting a previously unknown role for these cells in controlling transmission. Based on these findings, we sought to identify a signal(s) upstream of basophils that could result in these phenotypes.

Basophils have several surface receptors, including TLR2, TLR4, FcεRI, IL-3R, IL-5R, and IL-18R, that contribute to cell activation (reviewed in Refs. 10-12). Given that IL-18 is produced by dendritic cells and macrophages early in malaria (13, reviewed in Ref. 14) and is also released from damaged endothelial cells as an alarmin (reviewed in Ref. 15), the basophil IL-18R was of particular interest. Further, we previously reported that plasma IL-18 was significantly increased at day 4 PI in our mouse model, the same time point at which circulating basophils are significantly increased in *P. y. yoelii* 17XNL–infected mice (8). To determine whether basophil-dependent protection of the intestinal barrier is dependent on the basophil IL-18R, we infected *IL18r*^flox/flox^ × Basoph8 [basoIL-18R (−)] mice and control *IL18r*^flox/flox^ [basoIL-18R (+)] mice with *P. y. yoelii* 17XNL to examine malaria-induced intestinal permeability and parasite transmission to *A. stephensi*. Our data revealed that malaria-induced intestinal permeability was strikingly increased on a single day PI, day 4, in basoIL-18R (−) mice relative to basoIL-18R (+) mice. In addition, this time point was marked by significantly increased plasma cytokines and chemokines in basoIL-18R (−) mice relative to uninfected baseline, a pattern that was not evident in infected basoIL-18R (+) mice, suggesting that basophil IL-18R signaling coordinates changes in cytokines and chemokines with intestinal permeability at a precise early time point in malaria. Finally, transmission studies with basoIL-18R (−) and basoIL-18R (+) mice revealed a surprising reversal of the transmission phenotype observed in basophil-depleted versus nondepleted mice (9). In contrast with increased transmission from infected basophil-depleted mice to *A. stephensi*, we observed decreased transmission from infected basoIL-18R (−) mice relative to basoIL-18R (+) mice, suggesting that basophil-dependent control of parasite transmission likely involves multiple signals from these rare granulocytes.

## MATERIALS AND METHODS

### Mice

Mice with basophils lacking IL-18R were generated by crossing Basoph8 mice (Jackson Laboratory stock no. 017578, developed by R. Locksley [16]) with *IL18r*^flox/flox^ mice (provided by R. Flavell). A total of 108 basoIL-18R (−) mice were used as experimental animals, and 91 sex- and age-matched *IL18r*^flox/flox^ [basoIL-18R (+)] mice were used as controls. Experimental procedures were conducted on both male (*n* = 87) and female (*n* = 112) mice at 6–8 wk of age. Mice were housed in ventilated microisolator caging and provided food and water ad libitum. All procedures were approved by the Institutional Animal Care and Use Committee of the University of Idaho (protocol number IACUC- 2020-10, approved March 30, 2020).

### Mouse infection and monitoring

A total of 108 basoIL-18R (−) mice and 91 basoIL-18R (+) control mice were used across three replicates of time-course studies, six replicates of transmission studies, and one replicate of flow cytometry. Mice were infected as described previously (9, 17), with 172 mice injected i.p. with 150 μl of 1 × 10^6^ *P. y. yoelii* 17XNL–infected RBCs and 27 injected i.p. with 150 μl United States Pharmacopeia saline at day 0. Mice were sacrificed at 3 d PI (transmission studies only, when gametocytes are maximally infective to *A. stephensi*) or at 4, 6, 8, and 10 d PI for blood and/or tissue collection. Starting at 2 d PI, daily parasitemias were calculated from thin blood smears stained with Giemsa as described elsewhere (9). Gametocytemia at 3 d PI was calculated as the number of RBCs infected with gametocytes divided by the total number of RBCs counted in 25 fields viewed at 1000× magnification. Mice were monitored daily for weight loss and reduced activity to determine humane end points (>20% of starting weight, lack of feeding, drinking, and grooming). Blood samples were obtained by cardiac puncture for determination of bacterial 16S ribosomal DNA copies. Plasma samples were prepared from remaining blood and aliquoted for quantification of cytokines, chemokines, Mcpt1, Mcpt4, and IgE. Plasma aliquots were stored at −80° C until analysis. Ileum tissue was collected and snap frozen (~3 cm) for cytokine and chemokine analysis, with the remainder formalin-fixed for histological analysis.

### *In vivo* intestinal permeability

In all three replicates of the time-course studies, intestinal permeability was quantified as described previously (8, 9, 17). In brief, mice were orally gavaged with 4 kDa FITC dextran solution after a 4-h fast. Plasma was collected 3 h postgavage, and fluorescence was measured using a microplate reader (Molecular Devices LLC, San Jose, CA) at excitation/emission 490/520 nm.

### Ileum histochemistry and MC staining

Formalin-fixed ileum samples were prepared as described previously (8) and subjected to enzyme histochemical staining to identify naphthol AS-D chloroacetate esterase (NASDCE) activity (ref. 91C-1KT; Sigma-Aldrich, St. Louis, MO) according to the manufacturer’s instructions. For each mouse examined [*n* = 103; 55 basoIL-18R (−), 48 basoIL-18R (+)], MCs were enumerated in 50 high-power fields.

### ELISAs

Levels of Mcpt1 (eBioscience, San Diego, CA), Mcpt4 (Aviva Systems Biology, San Diego, CA), and IgE (eBioscience) were determined in plasma samples using commercial ELISAs and a microplate reader (BMG LABTECH, Cary, NC). IL-33 was measured in ileum tissue (prepared as in Ref. 17) using a Bio-Plex Pro Mouse Cytokine IL-33 Set (Bio-Rad, Hercules, CA) according to the manufacturer’s instructions.

### Extraction of DNA from blood and bacterial 16S DNA quantitative PCR

DNA was isolated from blood aliquots using the DNeasy Blood & Tissue kit (Qiagen, Germantown, MD) according to the manufacturer’s protocol. DNA was diluted to 4 ng/μl, and samples were assayed in triplicate using Maxima SYBR green/ROX qPCR master mix (2×) (Thermo Fisher Scientific, Waltham, MA) and 16S forward and reverse primers (forward 5′-ACTCCTACGGGAGGCAGCAGT-3′, reverse 5′-ATTACCGCGGCTGCTGGC-3′) at final concentrations of 0.4 μM. 16S copies were quantified using a standard curve as described previously (8). Data were analyzed using QuantStudio 6 Flex (Applied Biosystems, Waltham, MA).

### Cytokines and chemokines in plasma and ileum samples

Levels of plasma cytokines and chemokines (IL-1α, IL-1β, IL-2, IL-3, IL-4, IL-5, IL-6, IL-9, IL-10, IL-12p40, IL-12p70, IL-13, IL-17, eotaxin, G-CSF, GM-CSF, IFN-γ, KC, MCP-1, MIP-1α, MIP-1β, RANTES, TNF-α) were determined in two replicates of the time-course studies (*n* = 101) as described previously (8). Concentrations of cytokines and chemokines in 3 cm of ileum tissue were determined for mice from all three replicates of the time-course studies (*n* = 145). Ileum tissue was collected and processed as described previously (17).

### Transmission studies

Transmission studies were carried out as described previously (9, 17). In brief, *A. Stephensi* (Indian strain, reared as described in Ref. 18), were fed on 6- to 8-wk-old male or female basoIL-18R (−) mice (*n* = 23) or basoIL-18R (+) mice (*n* = 25) at 3 d PI. Mice were anesthetized with ketamine (50 mg/kg) and xylazine (5 mg/kg) and placed individually on top of a carton containing ~60 3- to 5-d-old female mosquitoes. Mosquitoes were allowed to feed for 15 min. After feeding was complete, mice were euthanized via CO_2_ asphyxiation followed by cervical dislocation. Non-blood-fed mosquitoes were removed from the cartons. At 10 d postfeeding, 25–35 mosquitoes per carton were dissected and stained with mercurochrome to quantify numbers of oocysts per midgut (infection intensity) and number of mosquitoes with at least one midgut oocyst out of the total fed mosquitoes (infection prevalence).

### Flow cytometry

To confirm depletion of basophil IL-18R (Supplemental Fig. 1), we collected spleens from basoIL-18R (−) mice (*n* = 3) and basoIL-18R (+) mice (*n* = 3) at 4 d PI for flow cytometry. Spleens were prepared as described previously (9). In brief, 10^7^ cells per spleen were incubated with anti-mouse CD49b (PerCP) (eBioscience), anti-mouse FCεR1α (allophycocyanin) (eBioscience), and anti-mouse IL-18R (PE) (eBioscience) at the manufacturer’s recommended concentrations for 1 h at room temperature, protected from light, then stained with DAPI (Invitrogen, Waltham, MA). Cells were counted using a CytoFLEX Flow Cytometer (Beckman Coulter, Brea, CA). Dead cells were excluded based on DAPI staining, and basophils within this population were defined as expressing both CD49b and FCεR1α (19, 20). Data were analyzed with CytExpert Software (Beckman Coulter).

### Statistical analyses

Parasitemia, intestinal permeability, bacterial 16S DNA copies per microliter of blood and Mcpt1, Mcpt4, IgE, MCs per high-power field, and cytokine/chemokine concentrations were analyzed by Robust Regression and Outlier Removal method (maximum false discovery rate Q = 1%) to exclude outliers. Nonnormal data were compared among time points using the Kruskal–Wallis test followed by Dunn’s multiple-comparison test of each time point between genotypes. Normally distributed data were analyzed using Brown–Forsythe & Welch ANOVA Mean oocysts per midgut and gametocytemia were compared using a Mann–Whitney *U* test. Infection prevalence was analyzed by Fisher’s exact test. The *p* values <0.05 for all analyses were considered significant.

### Ethics statement

All experiments were performed with the approval of the Institutional Animal Care and Use Committee of the University of Idaho (protocol number IACUC-2020-10, approved March 30, 2020).

## RESULTS

### *Basophil IL-18R deficiency had no effect on* P. y. yoelii *17XNL parasitemia*

**FIGURE 1.**
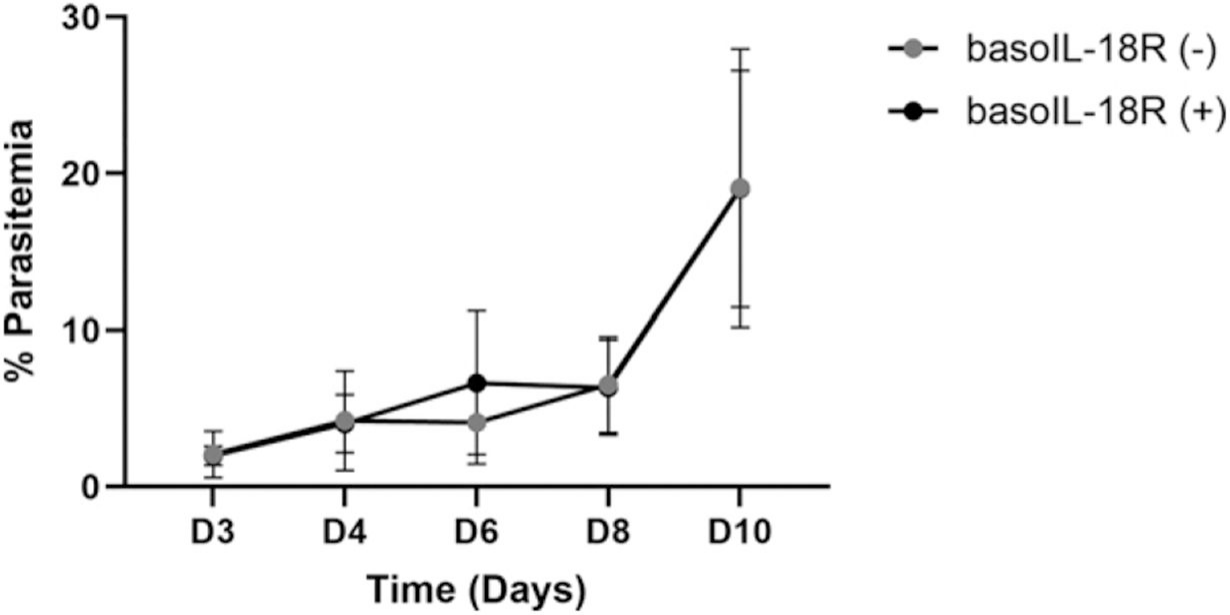
Peripheral parasitemia did not differ between *P. y. yoelii* 17XNL–infected basoIL-18R (−) mice (*n* = 52) and basoIL-18R mice (+) (*n* = 47) mice. Peripheral parasitemia was quantified as the number of RBCs infected with asexual parasites or gametocytes divided by the total number of RBCs counted in five fields viewed at 1000× magnification on a light microscope. Error bars represent means ± SDs. Data were analyzed using Brown–Forsythe and Welch ANOVA test followed by Dunnett’s T3 multiple comparison between genotypes at each time point. The *p* values <0.05 were considered significant.

Parasitemia in basoIL-18R (−) mice was not different at any time point relative to that in basoIL-18R (+) mice (Fig. 1). This pattern was consistent with that observed in basophil-depleted and nondepleted mice (9), suggesting that phenotypic differences between the two genotypes were not confounded by differences in parasite burden.

### Basophil IL-18R deficiency was associated with increased intestinal permeability at day 4 PI and ileal MC influx at day 10 PI

**FIGURE 2.**
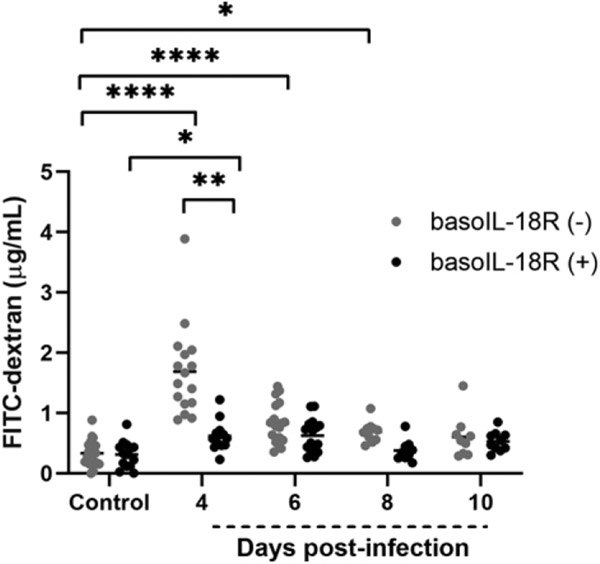
BasoIL-18R (−) mice show increased permeability to FITC-dextran at 4 d PI compared with basoIL-18R (+) mice and uninfected control mice. In vivo intestinal permeability quantified by plasma FITC-dextran concentration after oral gavage of *P. y. yoelii* 17XNL–infected and uninfected control mice of each genotype. Each dot represents a single mouse. Data were analyzed with Kruskal–Wallis test followed by Dunn’s multiple comparison between genotypes at each time point and between the respective uninfected controls. The *p* values <0.05 were considered significant. *p ≤ 0.05, **p ≤ 0.01, ****p ≤ 0.0001.

**FIGURE 3.**
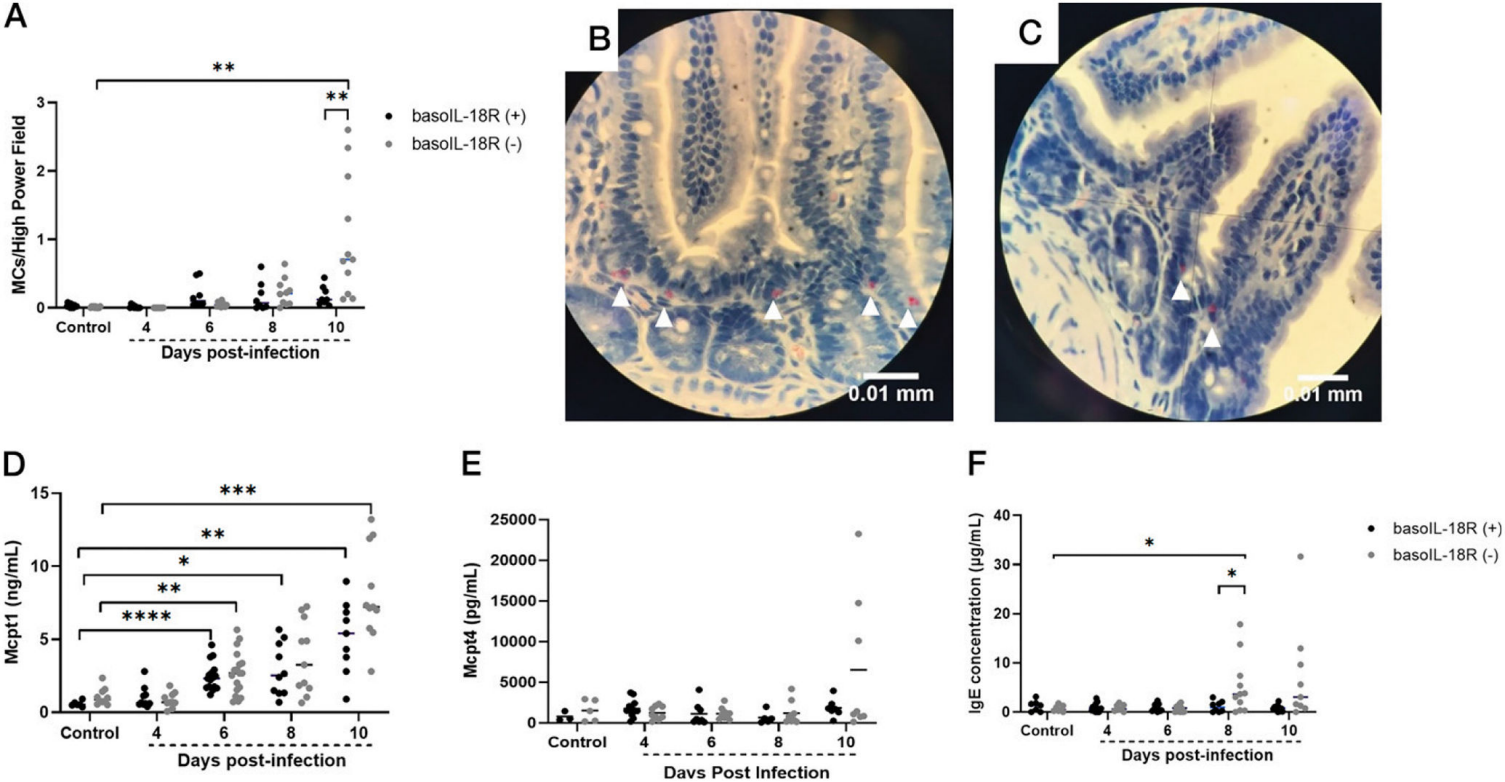
MC numbers in the ileum and MC-associated factors Mcpt1 and IgE increase in plasma of *P. y. yoelii* 17XNL–infected and uninfected control mice of each genotype, with significant differences in MC number at day 10 PI and IgE at day 8 PI. (**A**) Mean numbers of ileal MCs per high-powered field from NASDCE staining of ileal sections from infected and uninfected mice of both genotypes. Each dot represents one mouse. The data were analyzed by Welch’s *t* test between genotypes at each time point and the respective uninfected controls. The *p* values <0.05 were considered significant. **p ≤ 0.01. (**B** and **C**) Stained MCs (pink cells indicated by white arrowheads) in the ileum of a basoIL-18R (−) mouse (B) and a basoIL-18R (+) mouse (C) at 10 d PI. (**D**) Plasma Mcpt1 levels as determined by ELISA in control (uninfected) basoIL-18R (−) mice and in basoIL-18R (+) mice at indicated days PI in both genotypes. (**E**) Plasma Mcpt4 levels as determined by ELISA in control (uninfected) basoIL-18R (−) mice and in basoIL-18R (+) mice at indicated days PI in both genotypes. (**F**) Plasma IgE as determined by ELISA in control (uninfected) basoIL-18R (−) mice and in basoIL-18R (+) mice at indicated days PI in both genotypes. Data were analyzed with Brown–Forsythe and Welch ANOVA. The *p* values <0.05 were considered significant. *p ≤ 0.05, **p ≤ 0.01, ***p ≤ 0.001, ****p ≤ 0.0001.

Given our prior observations of MC-dependent intestinal permeability after *P. yoelii* infection (6, 7) and that basophil depletion was associated with increased intestinal permeability at 4, 6, and 8 d PI and increased ileal MC numbers at 8 d PI (9), we sought to assess the role of basophil IL-18R in these phenotypes. BasoIL-18R (−) mice exhibited a notable increase in intestinal permeability to FITC-dextran compared with basoIL-18R (+) mice at day 4 PI (Fig. 2), followed by a gradual return to baseline by 10 d PI. Interestingly, this peak in intestinal permeability coincides with a previously observed peak in circulating basophils in our model at 4 d PI (8), suggesting that basophil IL-18R contributes to protection against intestinal permeability at this time point. NASDCE staining of ileum sections revealed significantly increased MCs per field in basoIL-18R (−) mice relative to basoIL-18R (+) mice at 10 d PI (Fig. 3A-C). This observation was similar to findings in basophil-depleted mice, which showed significantly increased ileal MCs at day 8 PI relative to nondepleted mice (9). Patterns in both mutant mouse lines, therefore, were notably delayed relative to the infection-associated increase in ileal MCs in wild type mice at 4 d PI (8), suggesting that early malaria-induced MC influx into the ileum is at least partly dependent on basophils and signaling through IL-18R.

Because activated MCs can release Mcpt1 and Mcpt4 (reviewed in Ref. 21), proteases associated with increased intestinal permeability (22-25), we examined levels of these two proteases in plasma of infected and uninfected controls of each genotype. Levels of Mcpt1, produced largely by mucosal MCs (26), were significantly increased compared with uninfected mice in both genotypes at days 6, 8, and 10 PI, with no significant differences between genotypes at any time point (Fig. 3D). This pattern was also observed in basophil-depleted mice (9), suggesting that plasma Mcpt1 levels are not basophil dependent. Plasma levels of Mcpt4, largely a product of connective tissue MCs (21), were not significantly different from uninfected levels in either genotype at any time PI, despite a trend toward increased levels in basoIL-18R (−) mice at 10 d PI (Fig. 3E). We previously observed that *P. y. yoelii* 17XNL–infected wild type mice exhibited significantly increased plasma Mcpt4 levels at days 4 and 8 PI relative to uninfected controls (8), but this pattern was absent in infected, basophil-depleted mice relative to nondepleted control mice (9), suggesting that Mcpt4 synthesis is dependent on basophils and signaling through IL-18R Circulating IgE levels were significantly different between the two genotypes at day 8 PI, with levels in basoIL-18R (−) mice higher than uninfected baseline at this time point (Fig. 3F), potentially reflecting the observation that accumulation of IL-18 triggers IgE (27).

### *Basophil IL-18R deficiency had no effect on bacterial 16S DNA levels in blood during* P. y. yoelii *17XNL infection*

**FIGURE 4.**
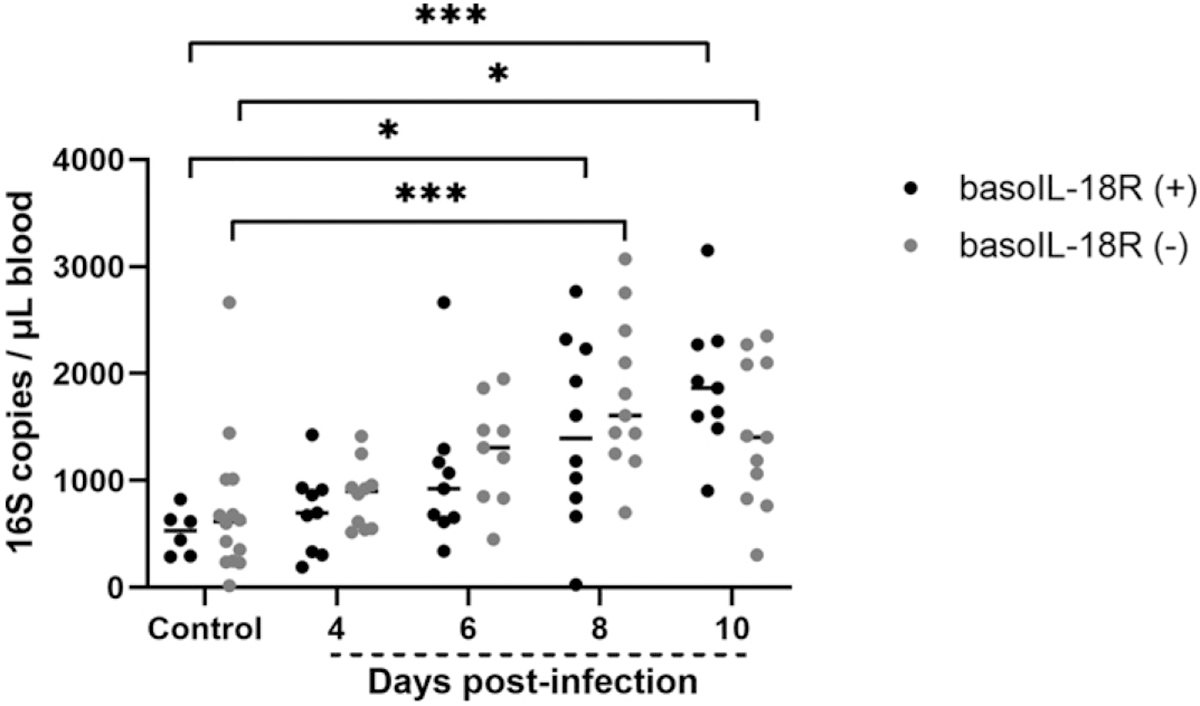
Bacterial 16S DNA copies in blood increased over the course of infection in *P. y. yoelii* 17XNL–infected mice but did not differ between genotypes. Bacterial 16S copies/μl blood in *P. y. yoelii* 17XNL–infected and uninfected control mice of each genotype. Each dot represents a single mouse. Data were analyzed with the Kruskal–Wallis test followed by Dunn’s multiple comparison between genotypes at each time point. The *p* values <0.05 were considered significant. *p ≤ 0.05, ***p < 0.001.

There were no differences in bacterial 16S DNA copies in blood between genotypes at any time point PI (Fig. 4). Similar to the findings in Céspedes et al. (8), blood 16S DNA copies in both basoIL-18R (−) mice and basoIL-18R (+) mice increased over the course of infection, becoming significantly elevated at days 8 and 10 PI (Fig. 4). These observations were similar to those for basophil-depleted and nondepleted mice, which exhibited rising blood 16S copies over the course of infection that did not differ between the genotypes at any time point (9).

### Basophil IL-18R deficiency was associated with reduced expression of specific type 1 and type 2 cytokines and chemokines in the ileum before and over the course of infoction

**FIGURE 5.**
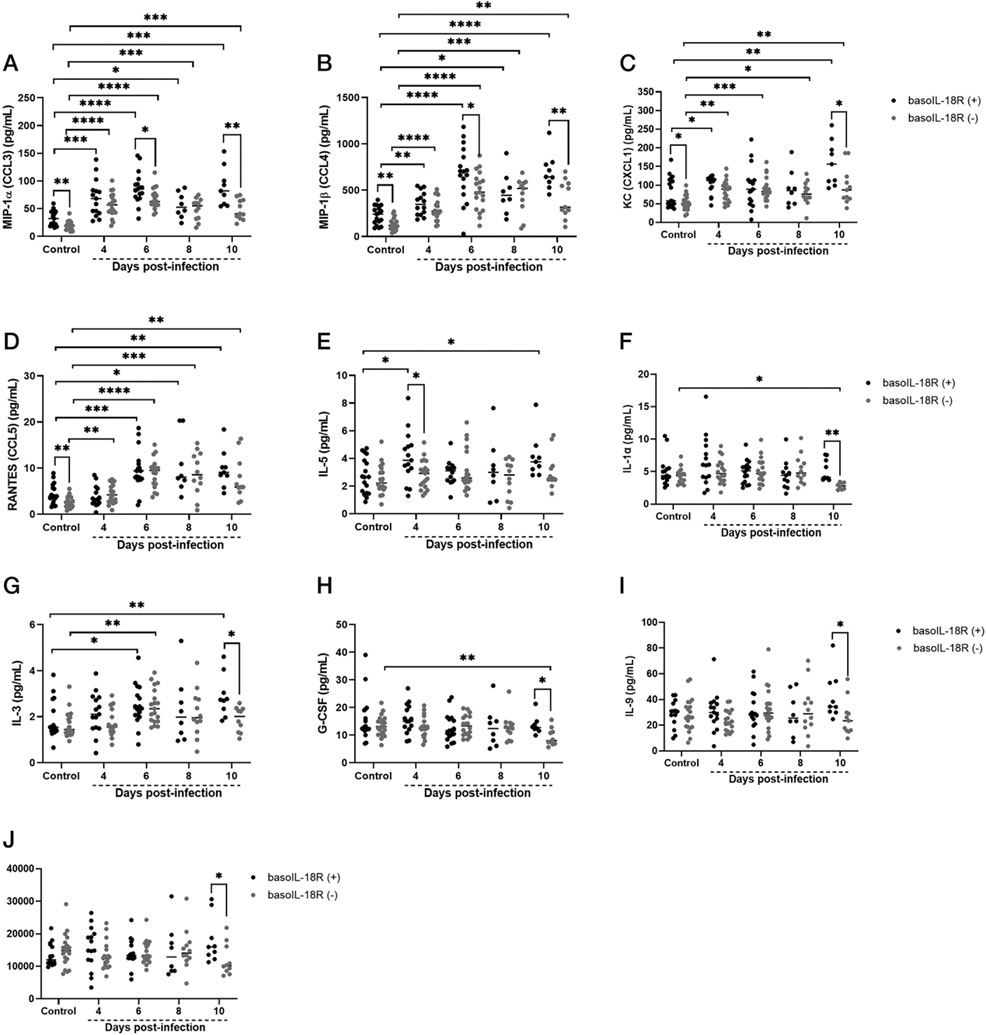
Ileal cytokines and chemokines in *P. y. yoelii* 17XNL–infected and uninfected control mice of each genotype. The *y*-axis represents the ileal concentrations of MIP-1α (CCL3) (**A**), MIP-1β (CCL4) (**B**), KC (CXCL1) (**C**), RANTES (CCL5) (**D**), IL-5 (**E**), IL-1α (**F**), IL-3 (**G**), G-CSF (**H**), IL-9 (**I**), and EOTAXIN (CCL11) (**J**). Each dot represents a single mouse. Normally distributed data (A, B, D, and I) were analyzed with the Brown–Forsythe & Welch ANOVA. Nonnormal data (C, E, F, H, and J) were analyzed with Kruskal–Wallis test followed by Dunn’s multiple comparison between the basoIL-18R (−) and basoIL-18R (+) mice at each time point and between infected and uninfected controls. The *p* values <0.05 were considered significant. *p ≤ 0.05, **p ≤ 0.01, ***p < 0.001, ****p ≤ 0.0001.

Levels of four chemokines, MIP-1α, MIP-1β, KC, and RANTES, showed genotype-specific differences relative to baseline and within time points (Fig. 5A-D). In all cases, levels of these chemokines were decreased in basoIL-18R (−) mice relative to basoIL-18R (+) mice (Fig. 5A-D), despite both genotypes showing increased levels of these chemokines with infection. Levels of six other chemokines and cytokines (IL-5, IL-1α, IL-3, G-CSF, IL-9, and eotaxin) were modestly increased or not increased with infection over time, but were reduced in basoIL-18R (−) mice relative to basoIL-18R (+) mice at 4 d PI (IL-5) or at 10 d PI (IL-1α, IL-3, G-CSF, IL-9, eotaxin; Fig. 5E-J). Levels of other cytokines and chemokines (IL-12p40, IL-1β, IL-4, MCP1, IL-10, IL-6) were not significantly different between genotypes at any time point PI, but rather were increased relative to uninfected genotypes at various times PI (Supplemental Fig. 2). Finally, the ileum cytokines IL-2, IL-12p70, IL-13, IL-17, GM-CSF, IFN-γ, TNF-α, and IL-33 remained unchanged relative to baseline over the course of infection in both genotypes (Supplemental Fig. 3).

### Basophil IL-18R deficiency was associated with increased levels of multiple type 1 plasma cytokines by 4 d PI

**FIGURE 6.**
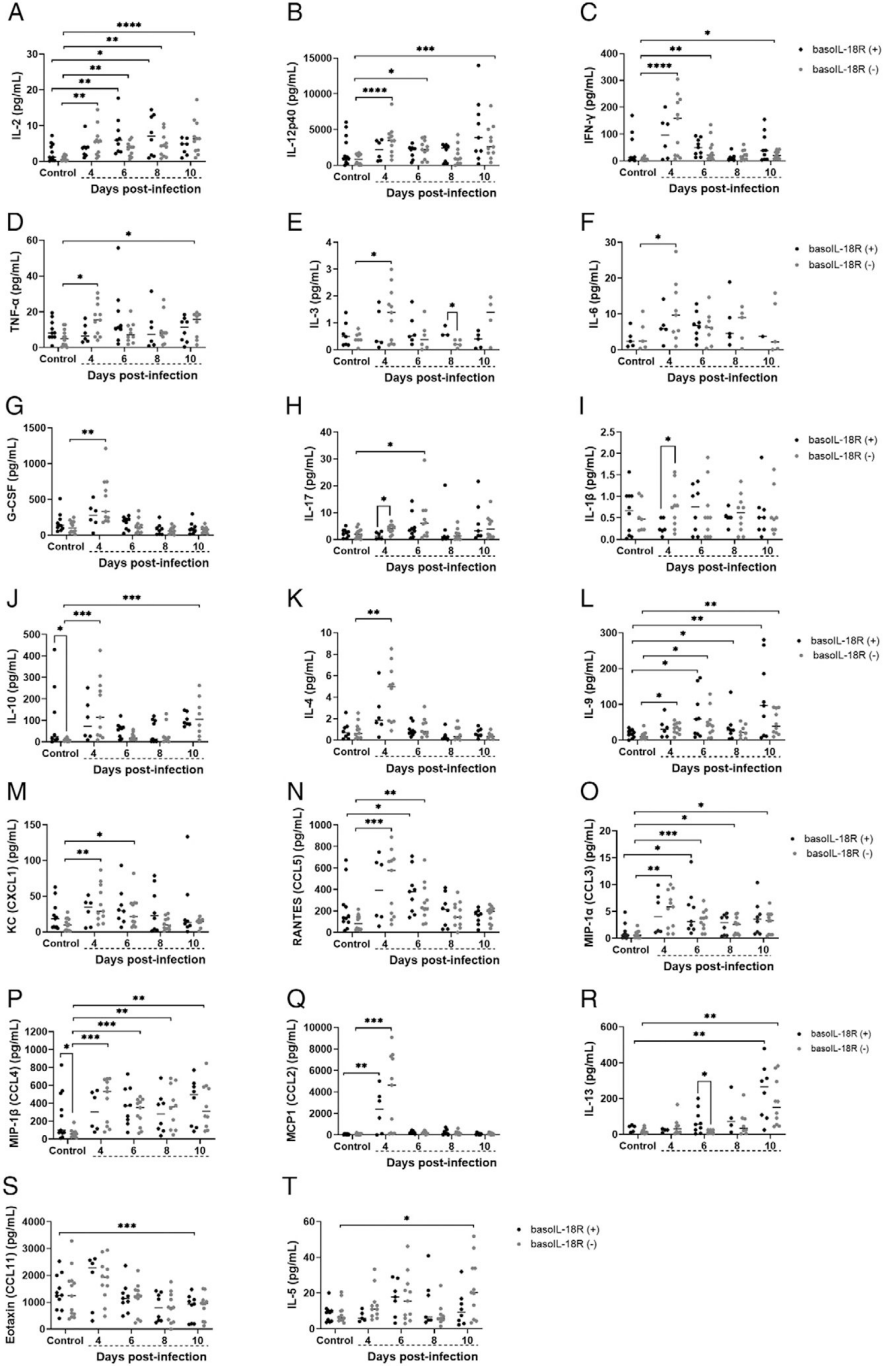
Plasma cytokines and chemokines in *P. y. yoelii* 17XNL–infected and uninfected control mice of each genotype. The *y*-axis represents the plasma concentrations of IL-2 (**A**), IL-12p40 (**B**), IFN-γ (**C**), TNF-α (**D**), IL-3 (**E**), IL-6 (**F**), G-CSF (**G**), IL-17 (**H**), IL-1β (**I**), IL-10 (**J**), IL-4 (**K**), IL-9 (**L**), KC (CXCL1) (**M**), RANTES (CCL5) (**N**), MIP-1α (CCL3) (**O**), MIP-1β (CCL4) (**P**), MCP1 (CCL2) (**Q**), IL-13 (**R**), eotaxin (CCL11) (**S**), and IL-5 (**T**). Each dot represents a single mouse. Normally distributed data (J-P, R, and S) were analyzed with the Brown–Forsythe & Welch ANOVA. Nonnormal data (A–I, Q, and T) were analyzed with Kruskal–Wallis test followed by Dunn’s multiple comparison between the basoIL-18R (−) and basoIL-18R (+) mice at each time point and between infected and uninfected controls. The *p* values <0.05 were considered significant. *p ≤ 0.05, **p ≤ 0.01, ***p < 0.001 ****p ≤ 0.0001.

BasoIL-18R (−) mice as early as day 4 PI exhibited increases in a number of type 1 cytokines, including IL-2 (Fig. 6A), IL-12p40 (Fig. 6B), IFN-γ (Fig. 6C), and TNF-α (Fig. 6D), relative to uninfected baseline levels, with some increases persisting through later time points. Increases in the same cytokines relative to baseline in basoIL-18R (+) mice were generally less frequent, with some exceptions. Specifically, IL-2, a cytokine required for regulatory T cell expansion in malaria (28, 29), was increased compared with baseline at day 4 PI, and this pattern persisted through day 10 PI in basoIL-18R (−) mice, while basoIL-18R (+) mice showed an increase above baseline only at days 6 and 8 PI (Fig. 6A). IL-12p40 and IFN-γ, important cytokines for malaria parasite clearance (reviewed in Ref. 30), were increased at days 4, 6, and 8 PI in basoIL-18R (−) mice, with no increase above baseline at any time PI in basoIL-18R (+) mice (Fig. 6B, 6C). TNF-α, a proinflammatory cytokine also associated with parasite clearance (31), was significantly increased from baseline at days 4 and 10 PI in baSOIL-I8R (−) mice, while no increases were observed in basoIL-18R (+) mice (Fig. 6D). Plasma IL-3 was increased above baseline at day 4 PI in basoIL-18R (−) mice but was later reduced relative to basoIL-18R (+) mice at 8 d PI (Fig. 6E). Plasma IL-6 and G-CSF were increased in basoIL-18R (−) mice at 4 d PI, with no increase at any time point in basoIL-18R (+) mice (Fig. 6F, 6G). Plasma IL-17 and IL-1β were significantly increased in basoIL-18R (−) mice relative to basoIL-18R (+) mice at 4 d PI, while IL-17 was elevated relative to baseline at 6 d PI only in basoIL-18R (−) mice (Fig. 6H, 6I). Notably, IL-10, IL-4, and IL-9 were also increased during infection (Fig. 6J-L). IL-10 increased only in basoIL-18R (−) mice at days 4 and 10 PI, although it was reduced in basoIL-18R (−) mice compared with basoIL-18R (+) mice at baseline (Fig. 6J). IL-4 was increased in basoIL-18R (−) mice only at day 4 PI (Fig. 6K). Plasma IL-9 was increased above baseline at days 4, 6, and 10 in basoIL-18R (−) mice, while levels in basoIL-18R (+) mice were increased relative to baseline at days 6, 8, and 10 PI (Fig. 6L). The neutrophil chemoattractant chemokine KC (32) was significantly increased in basoIL-18R (−) mice at days 4 and 6 PI, while no increases were observed in basoIL-18R (+) mice (Fig. 6M). Plasma RANTES was increased at 4 and 6 d PI above baseline in basoIL-18R (−) mice, but an increase above baseline in basoIL-18R (+) mice was observed only at 6 d PI (Fig. 6N). MIP-1α was elevated relative to baseline at days 4, 6, 8, and 10 PI in basoIL-18R (−) mice, while basoIL-18R (+) mice showed elevations relative to baseline only at day 6 PI (Fig. 6O). Similar to MIP-1α, levels of MIP-1β were increased relative to baseline at days 4, 6, 8, and 10 PI in basoIL-18R (−) mice (Fig. 6P). However, levels of MIP-1β were not increased relative to baseline at any time point PI in basoIL-18R (+) mice, although basoIL-18R (+) mice showed a small but significant increase in MIP-1β at baseline compared with basoIL-18R (−) mice (Fig. 6P). MCP1 was significantly increased above baseline in both genotypes only at 4 d PI, with a trend toward higher levels in basoIL-18R (−) mice (Fig. 6Q). Like plasma IL-10 (baseline; Fig. 6J) and IL-3 (day 8 PI; Fig. 6E), plasma IL-13 levels were significantly higher in basoIL-18R (+) mice relative to basoIL-18R (−) mice at day 6 PI with increased levels above baseline in both genotypes at day 10 PI (Fig. 6R). Eotaxin was the only cytokine that varied exclusively in basoIL-18R (+) mice, where it was decreased relative to baseline by day 10 PI (Fig. 6S). Plasma IL-5 was significantly increased above baseline only in basoIL-18R (−) mice and only at day 10 PI (Fig. 6T). There were no differences in levels of plasma IL-1α and IL-12p70 at any time point PI relative to baseline, nor were there differences in these cytokines between basoIL-18R (−) mice and basoIL-18R (+) mice at any time point (Supplemental Fig. 4).

### Transmission studies

**FIGURE 7.**
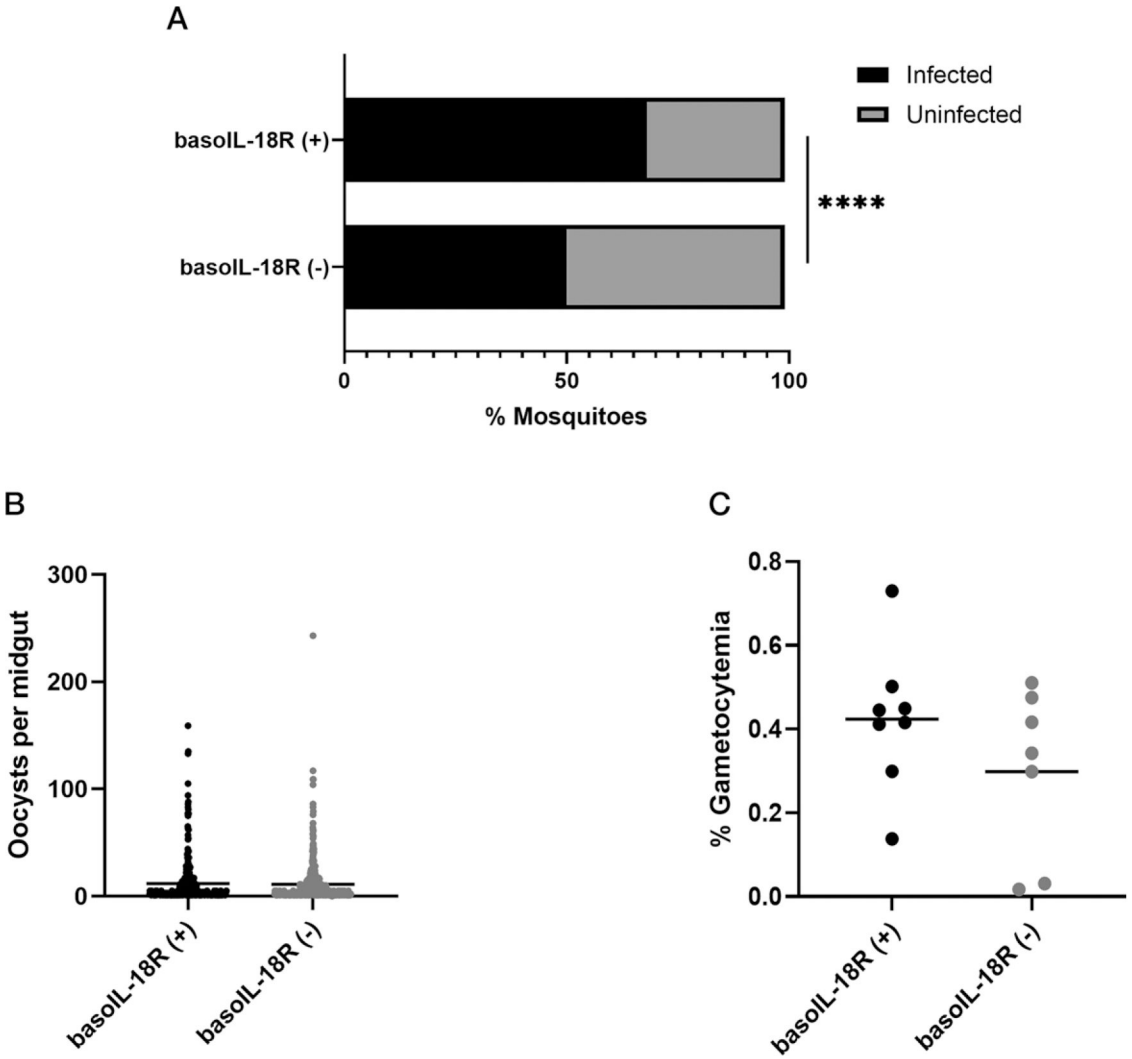
Basophil IL-18R depletion was associated with altered *P. y. yoelii* 17XNL transmission to *Anopheles stephensi*. (**A**) The prevalence of mosquito infection after feeding on basoIL-18R (−) mice and basoIL-18R (+) mice. Mosquitoes were counted as infected if they had at least one midgut oocyst. Data were analyzed by Fisher’s exact test, and *p* values <0.05843 were considered significant. ****p ≤ 0.0001. (**B**) *P. y. yoelii* 17XNL oocysts per midgut in infected *A. stephensi* after feeding on basoIL-18R (−) mice and basoIL-18R (+) mice at 3 d PI. Each dot represents one midgut with at least one oocyst. Data were analyzed using an unpaired *t* test with Welch’s correction. The *p* values <0.05 were considered significant. *p* = 0.2434. (**C**) Peripheral gametocytemia at day 3 (D3) PI in basoIL-18R (−) mice (*n* = 9) and basoIL-18R (+) mice (*n* = 9). Each dot represents one mouse. Data were analyzed by using an unpaired *t* test with Welch’s correction. *p* = 0.3969.

We have observed that mice depleted of basophils developed more gametocytes in circulation, and the mosquitoes that fed on these mice developed greater numbers of parasites relative to infected, nondepleted mice (9). Therefore, we sought to test whether the effects of basophils on gametocytemia and transmission could be attributed to basophil IL-18R To the contrary, however, the percentage of infected mosquitoes was *reduced* in mosquitoes that fed on basoIL-18R (−) mice relative to those that fed on basoIL-18R (+) mice (Fig. 7A), but there was no difference in infection intensity (oocysts per midgut) in mosquitoes that fed on mice with these genotypes (Fig. 7B). Gametocyte numbers trended lower in basoIL-18R (−) mice but were not significantly different between genotypes (Fig. 7C). These observations suggest that the basophil IL-18R regulates transmission biology, but further investigation is needed to identify the mechanisms by which basophils control gametocytemia and parasite transmission.

## DISCUSSION

Basophils are short-lived (~2.5 d) in circulation (33), have roles beyond allergy in fine-tuning host immune responses (34, 35), and are activated in both mouse and human malaria (8, 36). The studies herein affirm our observations (9) that basophils contribute to the maintenance of intestinal barrier integrity during malaria and extend these observations to include a role for basophil IL-18R. Although the mechanism by which this occurs remains to be elucidated, basophil-depleted mice and basoIL-18R (−) mice showed increased intestinal permeability from days 4–8 PI and at day 4 PI, respectively (9) (Fig. 2). Collectively, our data suggest for the first time, to our knowledge, that basophil IL-18R protects against early increases in intestinal permeability while other basophil factors regulate permeability later during infection.

Coinciding with the peak in intestinal permeability at day 4 PI, basoIL-18R (−) mice showed increases in 14 plasma cytokines and chemokines (Fig. 6A-G, J-P) compared with baseline, a pattern not observed in the basoIL-18R (+) mice. Six of these, IL-12p40, IFN-γ, TNF-α, IL-6, IL-4, and IL-9, have previously been demonstrated to cause degradation of tight junctions between epithelial cells and increased intestinal permeability in other disease models (reviewed in Refs. 37-40). IL-10, largely considered to be protective in maintaining intestinal barrier integrity (41, 42), was also increased in the plasma at days 4 and 10 PI compared with baseline in basoIL-18R (−) mice, but not in basoIL-18R (+) mice. Interestingly, IL-10 has also been shown to enhance MC responses mediated by IgE, and sensitized IL-10^−/−^ mice were protected from MC influx into the jejunum after OVA challenge (43). Increased levels of IL-4 in conjunction with IL-3, which can induce MC proliferation (44, 45, reviewed in Ref. 46), and IL-9, an important regulator of intestinal immunity and mucosal MCs (47) in basoIL-18R (−) mice, could help to explain increased MC numbers observed at 10 d PI in basoIL-18R (−) mice (Fig. 3A-C). KC, RANTES, MIP-1α, and MIP-1β, which were all significantly elevated in circulation at 4 d PI in basoIL-18R (−) mice (Fig. 6M-P), have also been implicated in the pathogenesis of inflammatory bowel disease (reviewed in Ref. 48), a condition associated with increased intestinal permeability (reviewed in Ref. 49). Systemic administration of MIP-1α, produced by many cell types at high levels in circulation during infection (50-53), markedly increased intestinal damage in a colitis model (54). Other studies have highlighted dichotomous roles for MIP-1α and MIP-1β in regulating systemic/type 1 and mucosal/type 2 host immune responses, respectively (55). These studies reported that MIP-1α treatment promoted robust production of IgG and IgM, while MIP-1β treatment promoted IgA and IgE production, in addition to lower levels of IgG and IgM (55). Interestingly, basoIL-18R (−) mice, which showed increases in circulating levels of MIP-1β beginning at day 4 PI and continuing through day 10 PI (Fig. 6P), also showed increased circulating IgE at 8 d PI (Fig. 3E), while basoIL-18R (+) mice, in which MIP-1β never increased above baseline levels, did not. Given that MCs express the FcεRI (56) and that binding of IgE to this receptor promotes their survival (57), this increase in circulating IgE at day 8 PI could contribute to the influx of MCs seen at day 10 PI in the baSOIL-I8R (−) mice (Fig. 3A-C). In addition, in parasitic infections, the proinflammatory cytokine and alarmin IL-1α is a strong coactivating stimulus for MCs, resulting in MC synthesis of IL-3, IL-5, IL-6, and IL-9, which can have autocrine and paracrine effects (IL-3, IL-9) on MCs (58). Synthesis of G-CSF can be induced by IL-1α (reviewed in Ref. 59) and is a master regulator of neutrophilic homeostasis, mobilization, and the release of neutrophil extracellular traps (60). MC reconstitution of C57BL/6 Kit^W-sh/W-sh^ mice has been associated with reduced G-CSF levels and restoration of neutrophils to wild type levels in these mutant mice (61), suggesting that elevated MCs (Fig. 3A) and reduced G-CSF levels at 10 d PI may be associated with enhanced neutrophilic responsiveness in basoIL-18R (−) mice and the downward trend in bacterial 16S DNA copies in these mice at 10 d PI relative to basoIL-18R (+) mice (Fig. 4). Together, these data suggest that basophil IL-18R protects against increases in intestinal permeability PI in part by regulating early production of inflammatory cytokines and chemokines, which may in turn protect against MC influx in the ileum.

Overall, basoIL-18R (−) mice showed increases from baseline in numerous plasma cytokines and chemokines, including IL-2 (Fig. 6A), INF-γ (Fig. 6C), TNF-α (Fig. 6D), IL-3 (Fig. 6E), IL-6 (Fig. 6F), G-CSF (Fig. 6G), IL-10 (Fig. 6J), IL-4 (Fig. 6K), KC (Fig. 6M), and MIP-1α (Fig. 6O) at day 4 PI that were absent in both basoIL-18R (+) mice and basophil-depleted mice (9). These unique increases in the plasma cytokines, many of which have been previously shown to regulate intestinal permeability, co-oc-cur with the peak of intestinal permeability at 4 d PI in our model. This suggests that basophil IL-18 signaling may be important for controlling the early inflammatory response to infection and promoting increased MC influx into the intestine later during infection. Many groups have noted instances where organisms that are genetic knockouts display no obvious phenotype, while knockdown organisms exhibit marked phenotypic changes (reviewed in Ref. 62). This observation has been attributed to multiple mechanisms of genetic compensation and/or transcriptional adaptation (reviewed in Ref. 62). Thus, although this phenomenon has not been studied in the context of basophil depletion, it is an important consideration when comparing findings across different strains of mutant mice. In addition, because the IL-18R on basophils is absent from birth, there is the possibility that there could be differential immune responses to infection. Nevertheless, given the consistencies in the major phenotypes of increased intestinal permeability and MC influx in both basoIL-18R (−) mice and basophil-depleted mice compared with their respective controls, we maintain that basophils protect against, rather than contribute to, malaria-induced damage to the intestinal barrier and play an important role in controlling the inflammation that underlies this increased permeability.

Our data have shown that increased intestinal permeability and ileal MC influx observed in basophil-depleted mice after *P. y. yoelii* 17XNL infection (9) were recapitulated in basoIL-18R (−) mice with minor temporal shifts, but the effect of basophil depletion on parasite transmission to *A. stephensi* was reversed in basoIL-18R (−) mice. That is, mosquitoes that fed on basoIL-18R (−) mice became infected less frequently than mosquitoes fed on infected basoIL-18R (+) mice, with no difference in intensity of infection (Fig. 7A, 7B). Plasma TNF-α and IFN-γ, both shown to have gametocidal properties (63-65), were significantly increased in basoIL-18R (−) mice close to the peak of transmission to mosquitoes (Fig. 6C, 6D). This may explain the trend toward decreased gametocytemia in basoIL-18R (−) mice relative to basoIL-18R (+) mice (Fig. 7C). However, more work is necessary to elucidate the mechanism(s) by which basophil-mediated control of transmission occurs, because these could have major implications for malaria-control efforts.

## Supplementary Material

Supplemental figures

## Abbreviations used in this article:

basoIL-18R (−): *IL18r*^flox/flox^ × Basoph8
basoIL-18R (+): control *IL18r*^flox/flox^
MC: mast cell
Mcpt1: mast cell protease 1
NASDCE: naphthol AS-D chloroacetate esterase
PI: postinfection
