# Supplementary material for: The Basophil IL-18 Receptor Precisely Regulates the Host Immune Response and Malaria-Induced Intestinal Permeability and Alters Parasite Transmission to Mosquitoes without Effect on Gametocytemia
Source: Immunohorizons. Author manuscript; Available in PMC 2023 Mar 1. (PMC9977167; doi:10.4049/immunohorizons.2200057)
Supplement: Supplemental figures [file NIHMS1870868-supplement-Supplemental_figures.docx]

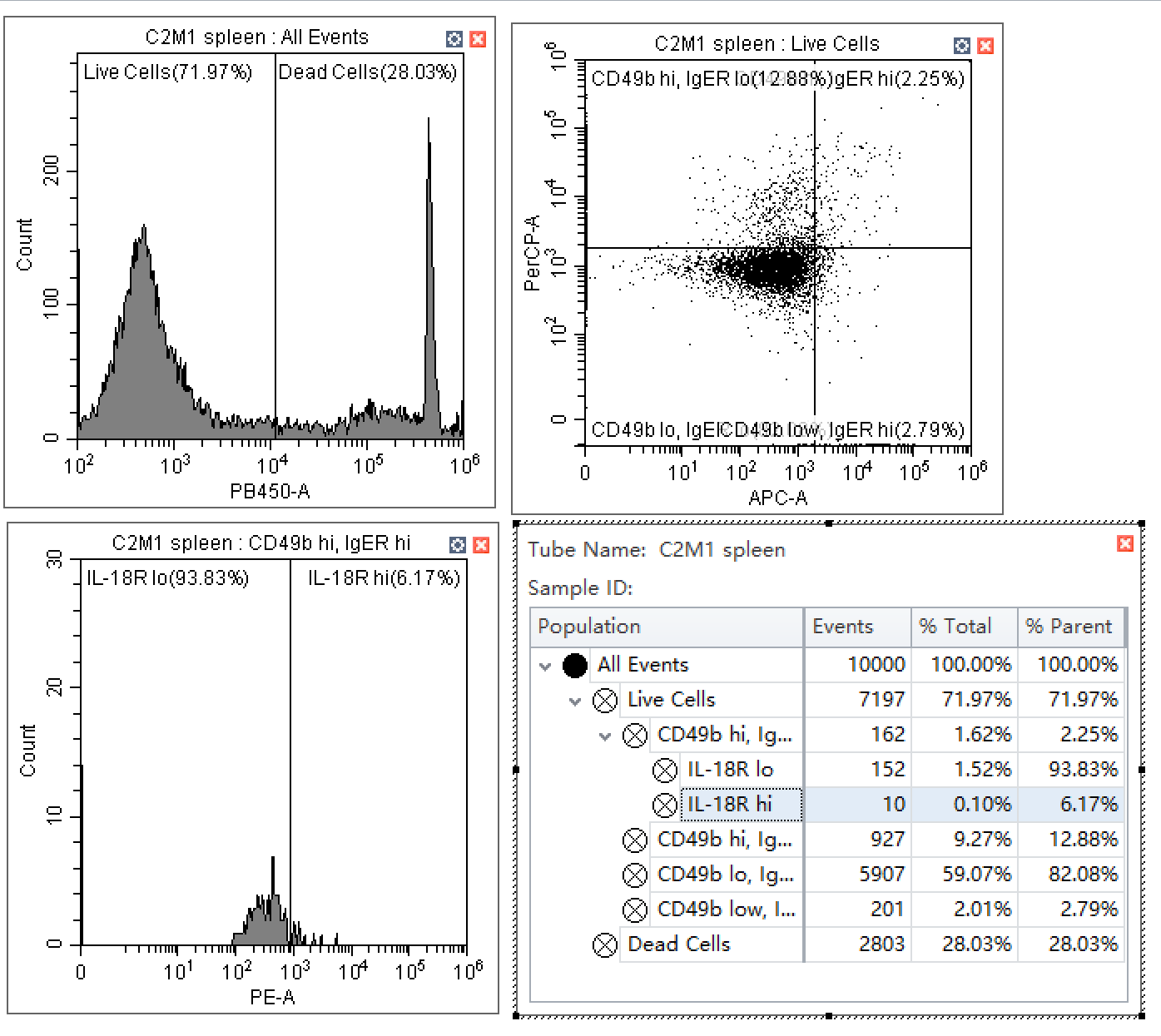

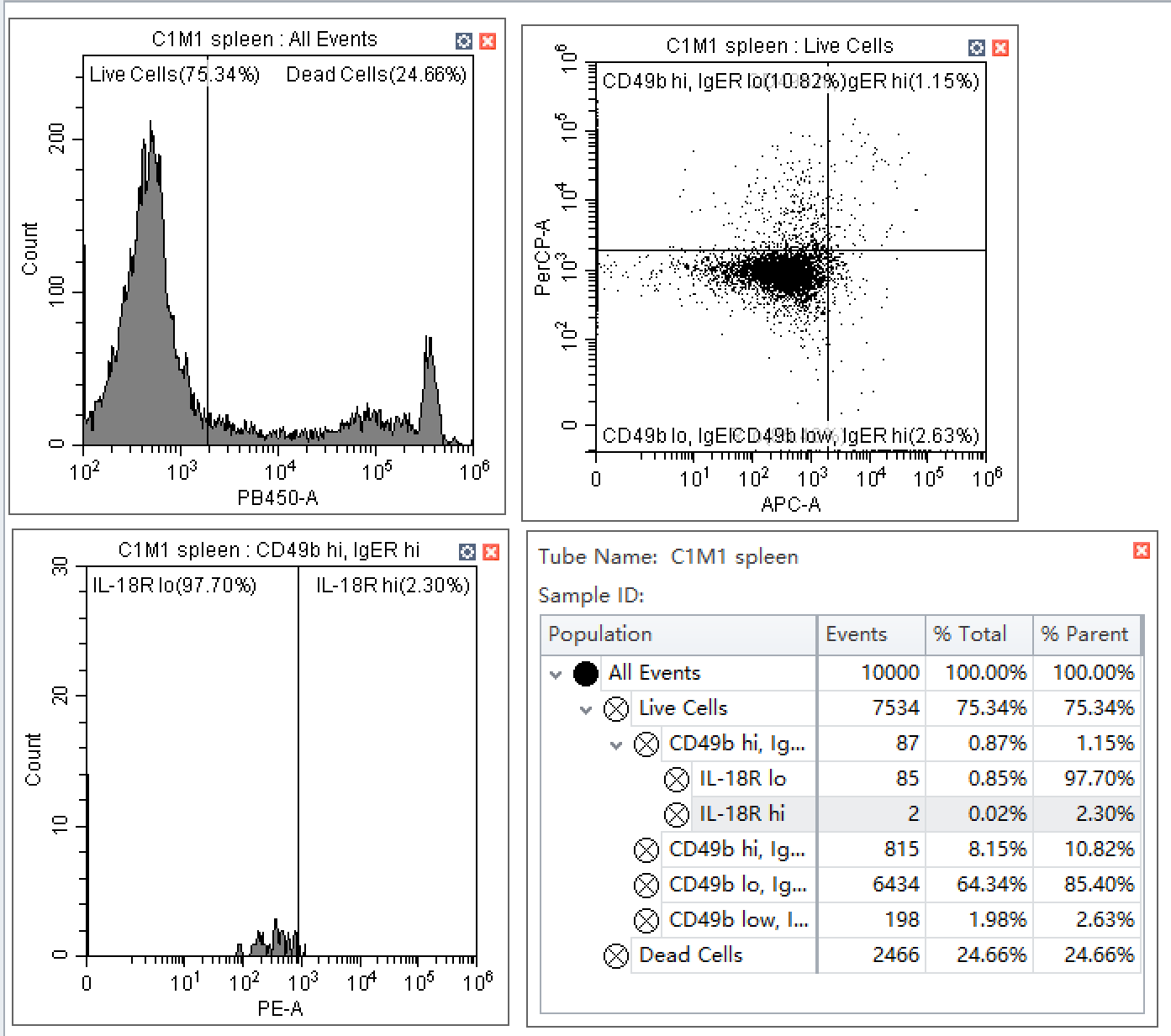


**B**

**A**

**Figure S1.** Flow cytometry gating strategy to confirm depletion of basophil IL-18R. After gating out dead cells (DAPI/PB450+), FCεRI (APC) and CD49b (PerCP) were used to define the basophil population in the spleen. The basophil population was then gated on high and low expression of IL-18R (PE) (**A&B**). (**A**) Representative plot from a mouse lacking IL-18R on basophils [basoIL-18 R (-)]. In the basoIL-18R (-) mouse, there were two IL-18R hi events in the basophil population. (**B**) Representative plot from a mouse with IL-18R on basophils [basoIL-18R (+)]. In the basoIL-18R (+) mouse, there were 10 IL-18R hi events in the basophil population.

**Figure S2**. Plasma Mcpt4 in *P. y. yoelii* 17XNL-infected and uninfected control mice of each genotype. Mcpt4 as determined by ELISA in control uninfected basoIL-18R (-) mice and basoIL-18R (+) mice and at indicated days post-infection in both genotypes. Data were analyzed with Kruskal-Wallis followed by Dunn’s multiple comparisons test. P values of <0.05 were considered significant.

**Figure S3**. Ileal cytokines and chemokines in *P. y. yoelii* 17XNL-infected and uninfected control mice of each genotype. The y-axis represents the ileal concentrations of IL-12p40 (**A**), IL-1β (**B**), IL-4 (**C**), MCP-1 (CCL2) (**D**), IL-10 (**E**) and IL-6 (**F**). Each dot represents a single mouse. Normally distributed data (C) were analyzed with the Brown-Forsythe & Welch ANOVA. Non-normal data (A, B, D-F) were analyzed with Kruskal-Wallis test followed by Dunn’s multiple comparison between the basoIL-18R (-) and basoIL-18R (+) mice at each time point, and between infected and uninfected controls. P values of < 0.05 were considered significant. *, P ≤ 0.05, **, P ≤ 0.01, ***, P < 0.001, ****, P ≤ 0.0001.

**Figure S4**. Ileal cytokines and chemokines in basoIL-18R (-) and basoIL-18R (+) mice at indicated days post-infection, and basoIL-18R (-) and basoIL-18R (+) uninfected controls. The y-axis represents the ileal concentrations of IL-2 (**A**), IL-12p70 (**B**), IL-13 (**C**), IL-17 (**D**), GM-CSF (**E**), IFN-γ (**F**), TNF-α (**G**), IL-33 (**H)**. Each dot represents a single mouse. Normally distributed data (**C**) were analyzed with the Brown-Forsythe & Welch ANOVA. Non-normal data (**A-B**, **D-H**) were analyzed with Kruskal-Wallis test followed by Dunn’s multiple comparison between the basoIL-18R (-) and basoIL-18R (+) mice at each time point, and between infected and uninfected controls. P values of < 0.05 were considered significant.

**Figure S5**. Plasma cytokines IL-1α and IL-12p70 do not change relative to baseline at any time point PI in basoIL-18R (-) and basoIL-18R (+) mice at indicated days post-infection, and basoIL-18R (-) and basoIL-18R (+) uninfected controls. The y-axis represents the plasma concentrations of IL-1α (**A**) and IL-12p70 (**B**). Each dot represents a single mouse. Normally distributed data (A) were analyzed with the Brown-Forsythe & Welch ANOVA. Non-normal data (**B**) were analyzed with Kruskal-Wallis test followed by Dunn’s multiple comparison between the basoIL-18R (-) and basoIL-18R (+) mice at each time point, and between infected and uninfected controls. P values of < 0.05 were considered significant.
